# Volume changes of diseased and normal areas in progressive fibrosing interstitial lung disease on inspiratory and expiratory computed tomography

**DOI:** 10.1007/s11604-024-01560-0

**Published:** 2024-04-06

**Authors:** Taiga Kobayashi, Yoshie Kunihiro, Takuya Uehara, Masahiro Tanabe, Katsuyoshi Ito

**Affiliations:** 1 Department of Radiology, Graduate School of Medicine, Yamaguchi University, 1-1-1 Minamikogushi, Ube, Yamaguchi 755-8505 Japan; 2 Department of Radiological Technology, Yamaguchi University Hospital, 1-1-1 Minamikogushi, Ube, Yamaguchi 755-8505 Japan

**Keywords:** Interstitial pneumonia, Interstitial lung disease, Expiratory CT, Volumetric measurement, Respiratory function

## Abstract

**Purpose:**

The diagnosis of progressive fibrosing interstitial lung disease (PF-ILD) using computed tomography (CT) is an important medical practice in respiratory care, and most imaging findings for this disease have been obtained with inspiratory CT. It is possible that some characteristic changes in respiration may be seen in normal and diseased lung in PF-ILD, which may lead to a new understanding of the pathogenesis of interstitial pneumonia, but it has never been examined. In this study, we collected and selected inspiratory and expiratory CT scans performed in pure PF-ILD cases, and evaluated the volumes of diseased and normal lung separately by manual detection and 3-dimensional volumetry to characterize the dynamic features of PF-ILD.

**Materials and methods:**

Cases were collected retrospectively from a total of 753 inspiratory and expiratory CT scans performed at our hospital over a 3-year period. Sixteen cases of pure PF-ILD, excluding almost all other diseases, were included. We measured their diseased, normal, and the whole lung volumes manually and evaluated the correlation of their values and their relationship with respiratory function tests (FVC, FVC%-predicted, and DLCO%-predicted).

**Results:**

The relative expansion rate of the diseased lung is no less than that of the normal lung. The “Expansion volume of total lung” divided by the “Expansion volume of normal lung” was found to be significantly associated with DLCO%-predicted abnormalities (p = 0.0073).

**Conclusion:**

The diseased lung in PF-ILD retained expansion capacity comparable to the normal lung, suggesting a negative impact on respiratory function.

## Introduction

Progressive fibrosing interstitial lung disease (PF-ILD) is often difficult to treat. In many patients with PF-ILD, the disease progresses irreversibly, eventually leading to respiratory failure of varying severities. Many findings have been reported regarding PF-ILD, with detailed studies of its characteristics conducted using computed tomography (CT). Using these findings to diagnose PF-ILD accurately is an important medical practice of daily respiratory care [1–4].

Also various reports have described the association of poor prognostic factors of fibrosing interstitial lung disease with respiratory function test items, such as the forced vital capacity (FVC)%-predicted [3, 5–7] and diffusing capacity of the lung for carbon monoxide (DLCO)%-predicted, imaging findings (e.g. degree of fibrosis, honeycombing, and traction bronchiectasis [3, 6, 8–14]), and epidemiology [7, 15]. However, information on these imaging findings is based on a static evaluation of inspiratory CT images. On the other hand, there have been several reports of evaluation using inspiratory and expiratory CT. These include an evaluation of effects of reconstruction algorithms on histogram parameters [16], air trapping [17], the degree of lung expansion [18], and the differences in lung motion between usual interstitial pneumonia (UIP) and nonspecific interstitial pneumonia (NSIP) [19]. As these studies show, registration during inspiratory and expiratory CT has become more accurate than before, but at this stage it is still difficult to detect positional changes and extract lesions simultaneously, and volume evaluation has not been performed separately for diseased and normal areas. However, there have been reports of evaluation of inspiratory CT by extracting diseased areas, and their relationship to disease progression is becoming clearer [20].

For elucidating the pathogenesis of PF-ILD, determining how respiration changes the volume of diseased and normal lung, and how they relate to each other in respiration may provide new insights; however, there have been no reports. In this study, we collected and selected inspiratory and expiratory CT scans performed in pure PF-ILD cases, and evaluated the volumes of diseased and normal lung separately by manual detection and 3-dimensional volumetry. Then, to characterize the dynamic features of PF-ILD, we evaluated the relationship between these volume changes on CT images and pulmonary function tests, and compared the rate of volume change between normal and diseased lung.

## Materials and methods

This study was approved by the institutional review board of our institution. The requirement for informed consent was waived owing to the retrospective study design.

### Patients

**Fig. 1:**
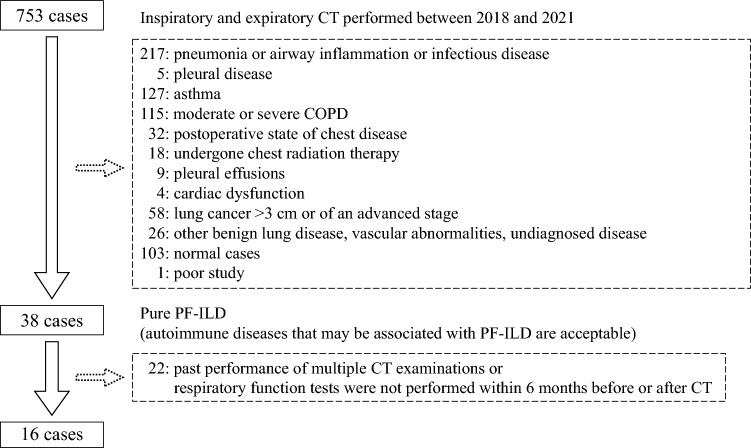
Selection of pure PF-ILD cases from among patients who had inspiratory and expiratory CT performed between 2018 and 2021. *CT* computed tomography, *COPD* chronic obstructive pulmonary disease, *PF-ILD* progressive fibrosing interstitial lung disease

Cases were retrospectively collected from among those that underwent inspiratory/expiratory CT between July 2018 and July 2021 (Fig. 1). Inspiratory/expiratory CT scans for evaluating pulmonary functional status were obtained from 753 patients with thoracic diseases hospitalized at our facility during the study period.

Of these 753 cases, the following diseases had already been clinically diagnosed; 217 had pneumonia, airway inflammation, or infectious disease; 5 had pleural disease or obvious pleural adhesions; 127 had asthma; 115 had moderate or severe chronic obstructive pulmonary disease (COPD); 32 was postoperative state of chest disease such as lung cancer; 18 had undergone chest radiation therapy for lung cancer or esophageal cancer or malignant lymphoma; 9 had pleural effusion; 4 had obvious cardiac dysfunction; 58 had lung cancer > 3 cm or of an advanced stage; and 26 had other benign lung disease, vascular abnormalities, or undiagnosed disease. As for COPD, referring to previous reports [21, 22], we decided to exclude cases in which emphysema clearly occupies more than 10% of the lung volume as moderate or severe. Therefore, the patients with PF-ILD were excluded when these abnormalities co-existed. In addition, there were 103 normal cases and 1 case with a poor study result due to poor breath holding. A total of 38 cases were diagnosed as PF-ILD, with no comorbidities affecting the respiratory function other than those associated with PF-ILD. We defined these cases as pure PF-ILD. The patients were clinically diagnosed with PF-ILD in terms of progressive fibrosis of imaging and worsening of respiratory function or symptoms. This case group is generally comparable to the criteria of previous report about PF-ILD [23], but some with more severe or mild pulmonary dysfunction are included. Among these cases, those with a respiratory function test performed within six months of CT were selected. The most recent test was selected if multiple tests had been performed. Thus, 16 patients were selected. In 13 of the 16 cases, the respiratory function tests were performed on the same day as the CT examinations being evaluated.

The patients consisted of 13 males and 3 females, with a mean age ± standard deviation (SD) of 74.2 ± 9.7 years old (Table 1). Seven patients had autoimmune diseases that might be related to PF-ILD (four with rheumatoid arthritis [RA], two with anti-neutrophil cytoplasmic antibody [ANCA]-associated vasculitides, and one with systemic scleroderma [SSc]). As for the radiological pattern, 14 cases were diagnosed to be UIP pattern and 2 cases were diagnosed to be an alternative diagnosis (1 case was suggested to be NSIP pattern and 1 case was suggested to be fHP (fibrotic hypersensitivity pneumonitis) pattern) based on previously established criteria [4, 24].

**Table 1:** Patient characteristics

N	16
Age, years	74.2 ± 9.7
Male:female	13:3
Autoimmune diseases	RA 4 ANCA-associated vasculitides 2 SSc 1 None 9
Radiological pattern	UIP 14 NSIP 1 fHP 1
FVC%-predicted normal:abnormal	11:5
DLCO%-predicted normal:abnormal (N = 14)	7:7
FVC%-predicted or DLCO%-predicted both are normal:at least one of which is abnormal	5:11

Ages are given as the mean ± standard deviation

*RA* rheumatoid arthritis, *ANCA* anti-neutrophil cytoplasmic antibody, *SSc* systemic scleroderma, *UIP* usual interstitial pneumonia, *NSIP* nonspecific interstitial pneumonia, *fHP* fibrotic hypersensitivity pneumonitis, *FVC* forced vital capacity, *DLCO* diffusing capacity of the lung for carbon monoxide

The 16 total patients were divided into 2 groups for each comparison of respiratory function test findings: “FVC%-predicted < 80% (abnormal)” vs. “FVC%-predicted ≥ 80% (normal),” “DLCO%-predicted < 80% (abnormal)” vs. “DLCO%-predicted ≥ 80% (normal),” and “FVC%-predicted or DLCO%-predicted is abnormal” vs. “both are normal”. However, since the DLCO%-predicted was not determined in 2 of the 16 patients, the comparison was performed in just 14 patients, excluding those 2 as for the DLCO%-predicted evaluation. Since both of these cases had an FVC%-predicted of < 80%, and both were classified as abnormal in the “FVC%-predicted or DLCO%-predicted” group, it was judged that the results would not be affected, and all 16 cases were evaluated in the “FVC%-predicted or DLCO%-predicted” group. In the above groupings, the FVC%-predicted was abnormal and normal in five and eleven cases, respectively; the DLCO%-predicted was abnormal and normal in seven and seven cases, respectively, and the FVC%-predicted or DLCO%-predicted was abnormal or both were normal in eleven and five cases, respectively.

### CT

CT was performed using a normal-resolution (NR) mode with a 0.5 mm × 80 detector configuration (Aquilion Precision; Canon Medical Systems Corporation, Tochigi, Japan) without intravenous contrast medium injection. CT images were reconstructed with a 1-mm slice thickness and a 512 × 512 matrix. The scanning parameters were 120 kVp and automated mA (expiratory CT; 100 mA). Monitors were used to view the lung images (window width = 1500 HU; window level = − 600 HU). We used the SYNAPSE VINCENT medical imaging system (version 6.3; Fujifilm Medical Co., Ltd., Tokyo, Japan) for the three-dimensional (3D) measurements of the lungs.

### Image analyses

**Fig. 2:**
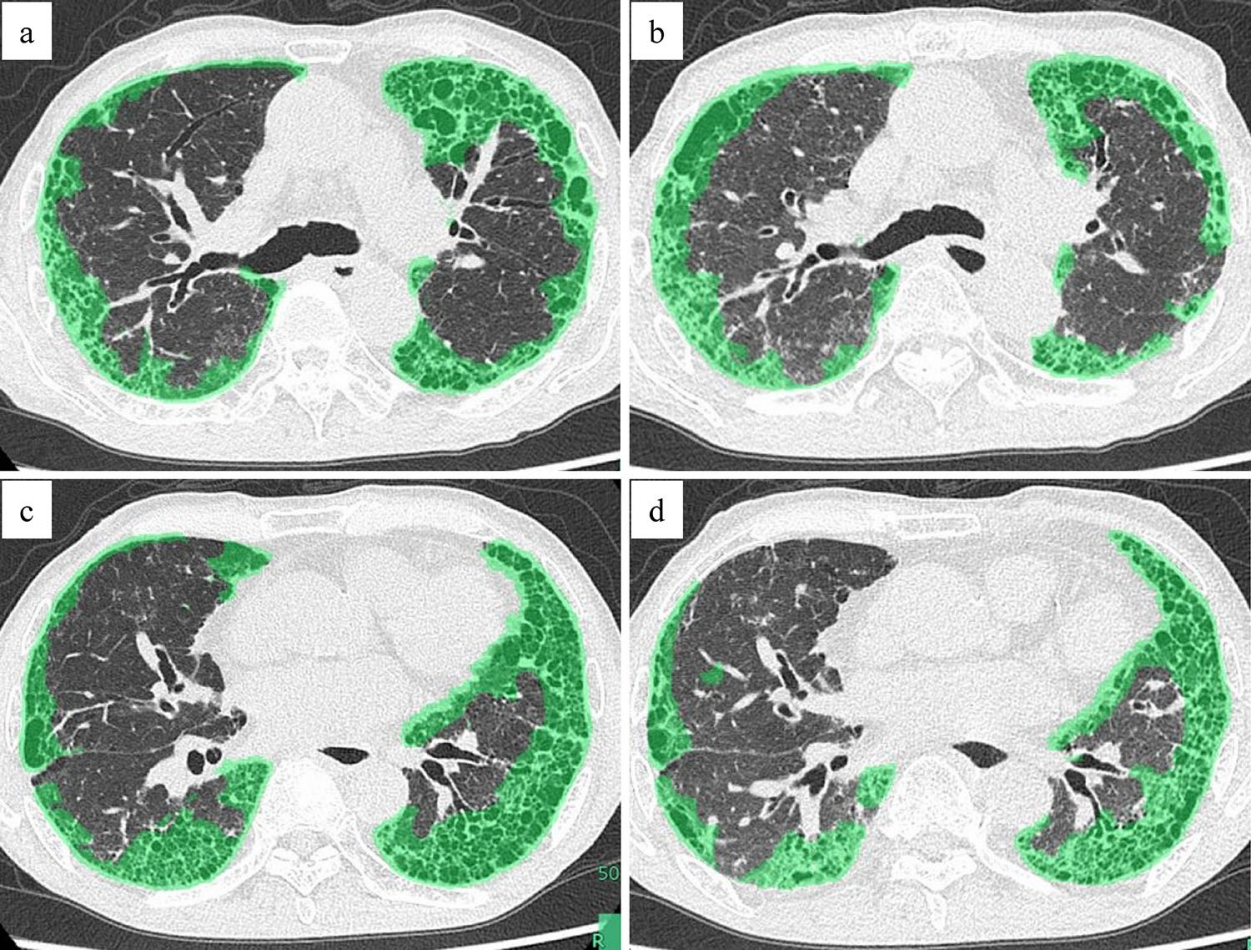
Examples of diseased lung measurements in inspiratory and expiratory CT. Inspiratory and expiratory CT images of a 79-year-old man. The area determined to be the diseased lung was manually selected for each 1-mm-thick slice (green area in the image). The volume of the diseased lung appears to change with respiration. Inspiratory CT at the level of the tracheal bifurcation (**a**). Expiratory CT at the level of the tracheal bifurcation (**b**). Inspiratory CT at the level of the left atrium (**c**). Expiratory CT at the level of the left atrium (**d**). *CT* computed tomography

CT images were evaluated by two thoracic radiologists with 16 and 22 years of experience, respectively (the authors). We discussed and determined how far each area extended and extracted the target areas in CT. In the 1-mm-thick images of both inspiratory and expiratory CT, the area within the entire thorax, excluding the mediastinum was extracted as the lung area for each slice. The diseased area was defined as honeycomb, reticulation, and if areas of GGA were clearly contiguous with a fibrotic lesion in a 1-mm-thick slice of inspiratory CT, they were also considered the fibrotic lesion and extracted. As the appearance of the honeycomb is greatly affected by the size of the cyst and the CT slice thickness [25], we decided to include all kinds of lesions, attempting to minimize the influence of imaging conditions, image quality conditions, and the evaluator. First, the target areas were extracted manually at each 1-mm-thick slices of axial section, so that all lesions in the series are included. Then, the lesions were repeatedly checked by moving back and forth throughout the series, finding lesions that extended beyond the margins and removing them manually in 1-mm-thick slices. The diseased areas on inspiratory and expiratory CT were extracted by matching vessel location and morphologic features checking the upper and lower slices to extract the same areas as possible (Fig. 2). The volume of the areas extracted in this way was calculated using the volumetric measurement function of the workstation. And the volume of the diseased lung was subtracted from the volume of the total lung to obtain the volume of the normal lung.

**Fig. 3:**
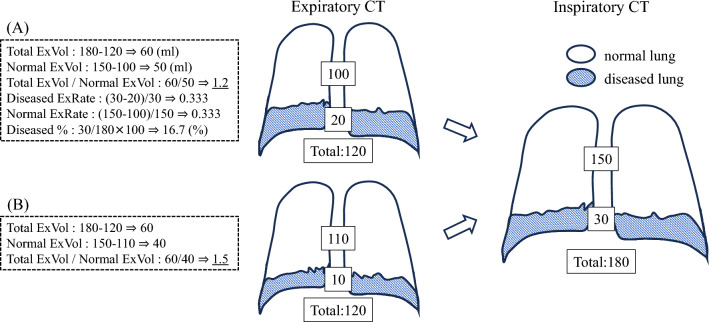
**A** An example schematic illustration for each value calculated in this study. The numbers in the picture represent the volume on the CT image. **B** Changes in Total ExVol/Normal ExVol with respiration. Two patients with identical inspiratory CT images could have different Total ExVol/Normal ExVol ratios if the expiratory CT images differed. *CT* computed tomography, *Total ExVol* expansion volume of total lung, *Normal ExVol* expansion volume of normal lung, *Diseased ExRate* expansion rate of diseased lung, *Normal ExRate* expansion rate of normal lung, *Diseased %* percentage of diseased lung in the total lung on inspiratory computed tomography

The items measured were the normal, diseased, and total lung volume on both inspiratory and expiratory CT. Based on this, the following values were calculated (Fig. 3):

- Total ExVol (Expansion volume of total lung) = total lung volume on inspiratory CT − total lung volume on expiratory CT (ml)
- Normal ExVol (Expansion volume of normal lung) = volume of normal lung on inspiratory CT − volume of normal lung on expiratory CT (ml)
- Diseased ExRate (Expansion rate of diseased lung) = (volume of diseased lung on inspiratory CT − volume of diseased lung on expiratory CT)/volume of diseased lung on inspiratory CT (ratio)
- Normal ExRate (Expansion rate of normal lung) = (volume of normal lung on inspiratory CT − volume of normal lung on expiratory CT)/(volume of normal lung on inspiratory CT) (ratio)
- Diseased % (percentage of diseased lung in the total lung on inspiratory CT) = (volume of diseased lung on inspiratory CT/total lung volume on inspiratory CT) × 100 (%)

Based on these values, we statistically examined the following items: correlation between the Total ExVol and FVC, correlation between the Diseased ExRate and Normal ExRate, correlation between the Diseased % and Normal ExRate, a comparison between the Diseased % and abnormality in respiratory function tests (FVC%-predicted, DLCO%-predicted, FVC%-predicted or DLCO%-predicted), and a comparison between the Total ExVol/Normal ExVol and abnormalities in respiratory function tests (FVC%-predicted, DLCO%-predicted, FVC%-predicted or DLCO%-predicted).

### Statistical analysis

The FVC and Total ExVol, Normal ExRate and Diseased ExRate, Normal ExRate and Diseased % were compared using Spearman’s correlation analysis, while the Diseased ExRate and Normal ExRate were compared using the Wilcoxon signed-rank test. The Diseased %, Total ExVol/Normal ExVol were compared between the normal and abnormal lung function groups (FVC%-predicted, DLCO%-predicted, FVC%-predicted or DLCO%-predicted) using the Mann–Whitney U test. A p-value < 0.05 was considered indicative of a significant difference. All statistical analyses were performed using the JMP^®^ Pro 16 software program (SAS Institute Inc., Cary, NC, USA).

## Results

**Fig. 4:**
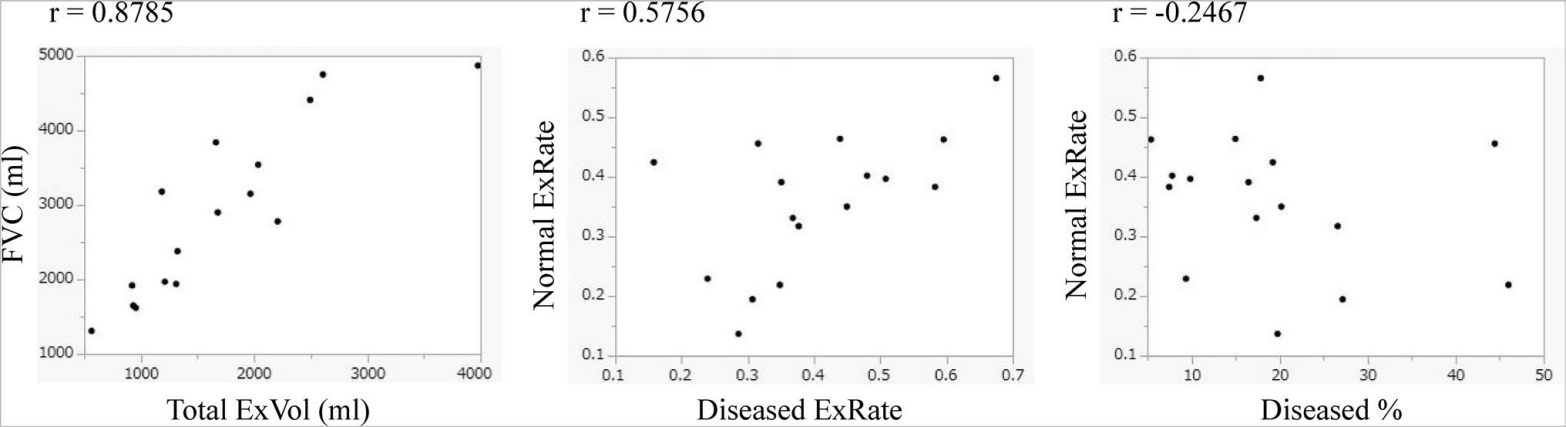
The correlation between the FVC and Total ExVol, Normal ExRate and Diseased ExRate, Normal ExRate and Diseased % using Spearman’s correlation analysis. The correlation coefficients of each value were as follows: FVC and Total ExVol (r = 0.8785), Normal ExRate and Diseased ExRate (r = 0.5756), and Normal ExRate and Diseased % (r = − 0.2467). *FVC* forced vital capacity, *Total ExVol* expansion volume of total lung, *Normal ExRate* expansion rate of normal lung, *Diseased ExRate* expansion rate of diseased lung, *Diseased %* percentage of diseased lung in the total lung on inspiratory computed tomography

The measurement results for each area on inspiratory and expiratory CT and the values of the respiratory function tests are shown in Table 2. The mean values of the Total ExVol, Normal ExVol, Diseased ExRate, Normal ExRate, Diseased %, and Total ExVol/Normal ExVol are shown in Table 3, and the correlations between the FVC and Total ExVol, Normal ExRate and Diseased ExRate, Normal ExRate and Diseased % using Spearman's correlation analysis are shown in Fig. 4. A strong correlation was detected between the FVC and Total ExVol (r = 0.8785). The mean Total ExVol was 1689.6 ml accounted for 58.5% of the FVC. The total lung volume on expiratory CT was 62.5% of that on inspiratory CT. A moderate correlation was found between the Diseased ExRate and Normal ExRate (r = 0.5756), and there was no significant difference between the Diseased ExRate and Normal ExRate (p = 0.0934), and in 12 of the16 cases, the Diseased ExRate was greater than the Normal ExRate. In addition, there was a slight negative correlation between the Diseased % and Normal ExRate (r = − 0.2467).

**Table 2:** Results of respiration function tests and volumetric measurements of inspiratory and expiratory CT

Respiratory function	
FVC%-predicted	89.9 ± 21.0%
DLCO%-predicted	78.6 ± 32.9%
FVC	2888.1 ± 1148.2 ml
Total lung volume on inspiratory CT	4503.8 ± 1277.3 ml
Volume of diseased lung on inspiratory CT	855.1 ± 546.7 ml
Volume of normal lung on inspiratory CT	3648.7 ± 1261.8 ml
Total lung volume on expiratory CT	2814.2 ± 719.5 ml
Volume of diseased lung on expiratory CT	524.4 ± 383.0 ml
Volume of normal lung on expiratory CT	2289.8 ± 723.1 ml

Values are given as the mean ± standard deviation

*FVC* forced vital capacity, *DLCO* diffusing capacity of the lung for carbon monoxide, *CT* computed tomography

**Table 3:** Mean values of the Total and Normal ExVol, Diseased and Normal ExRate, Diseased %, and Total ExVol/Normal ExVol

Total ExVol	1689.6 ± 853.2 ml
Normal ExVol	1358.9 ± 759.0 ml
Diseased ExRate	0.40 ± 0.14
Normal ExRate	0.36 ± 0.11
Diseased %	19.4 ± 12.0%
Total ExVol/Normal ExVol	1.33 ± 0.32

Values are given as the mean ± standard deviation

*Total ExVol* expansion volume of total lung, *Normal ExVol* expansion volume of normal lung, *Diseased ExRate* expansion rate of diseased lung, *Normal ExRate* expansion rate of normal lung, *Diseased %* percentage of diseased lung in the total lung on inspiratory computed tomography

**Fig. 5:**
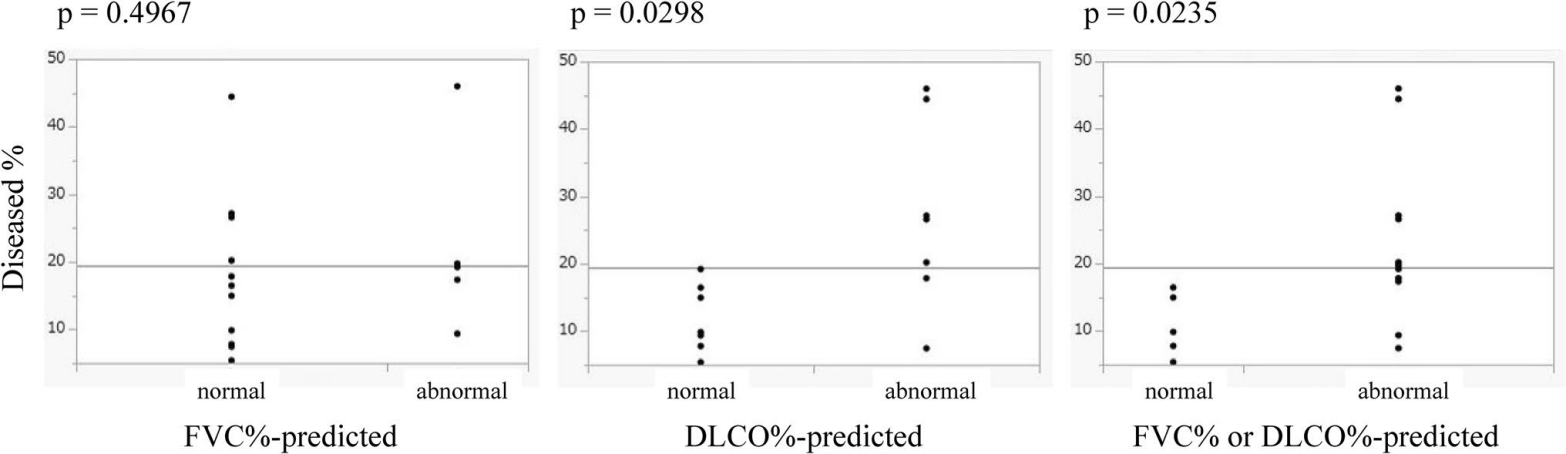
The results of comparing the Diseased % between the normal and abnormal lung function groups using the Mann–Whitney U test. There was a significant difference in the DLCO%-predicted abnormality (p = 0.0298), and FVC%-predicted or DLCO%-predicted abnormality (p = 0.0235). *Diseased %* percentage of diseased lung in the total lung on inspiratory computed tomography, *FVC* forced vital capacity, *DLCO* diffusing capacity of the lung for carbon monoxide

Figure 5 shows the results of comparing the Diseased % between the normal and abnormal lung function groups using the Mann–Whitney U test. There was a significant difference in the DLCO%-predicted abnormality (p = 0.0298), FVC%-predicted or DLCO%-predicted abnormality (p = 0.0235).

**Fig. 6:**
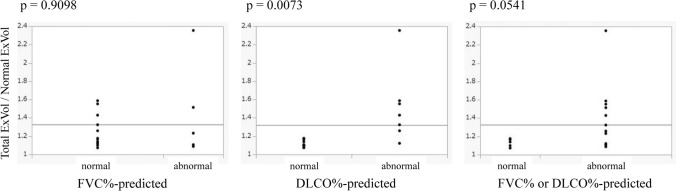
The results of comparing the Total ExVol/Normal ExVol between the normal and abnormal lung function groups using the Mann–Whitney U test. There was a significant difference in the DLCO%-predicted abnormality (p = 0.0073). *Total ExVol* expansion volume of total lung, *Normal ExVol* expansion volume of normal lung, *FVC* forced vital capacity, *DLCO* diffusing capacity of the lung for carbon monoxide

Figure 6 shows the results of comparing the Total ExVol/Normal ExVol between the normal and abnormal lung function groups using the Mann–Whitney U test. There was a significant difference in the DLCO%-predicted abnormality (p = 0.0073).

## Discussion

FVC and Total ExVol were strongly correlated and the accuracy of CT examination and volume measurement seemed to be assured (r = 0.8785). The total lung volume on expiratory CT was 62.5% of that on inspiratory CT, and the volume change during respiration accounted for 58.5% of the FVC. Based on the table of previous reports performed on healthy subjects [26], the reported value for the volume of expiratory/inspiratory imaging is 62.7% (2688.0/4286.2 ml), which is 44.9% (1598.2/3560 ml) of that in comparison with the FVC. Considering the difference in the number of subjects and in the target population, we believe that the difference between our study and theirs is reasonable.

Significant differences were found for Diseased % and presence of respiratory dysfunction. Previous reports have examined the association between the amount of fibrosis and prognosis [8], and our study suggested that it may also be related to respiratory function.

This study demonstrated that the relative expansion rate of the diseased lung is no less than that of the normal lung and that pulmonary injury caused by PF-ILD has limited effect on the relative expansion capacity of the lung. Johkoh et al. reported that the diameter of many honeycombs changes with respiration and attributed this to the continuity of the honeycomb with the airways in pathology [27]. In our volumetric measurements, there was a positive correlation between the Diseased ExRate and Normal ExRate (r = 0.5756), and there was no significant difference between the Diseased ExRate and Normal ExRate (p = 0.0934). This result seems natural, as the degree of inspiration affects them simultaneously, and this means that diseased lung still has the ability to expand comparable to the normal lung. In other words, the volume of diseased lung is smaller than normal lung during expiration because it is strongly collapsed, and from the state, it expands at a comparable rate to that of normal lung, so the volume on inspiratory CT and the amount of changing volume with respiration are smaller than those of the normal lung. This may be a characteristic of volume reduction in PF-ILD. Although the diseased lung presumed to be inherently less expanded than normal lung, they may be well expanded because they are located in the lower lobes and peripheral areas, which are more easily expanded by inspiration. Rather, another feature of PF-ILD may be the well expansion of the diseased lung because of their peripheral distribution. As a previous report have shown that fibrotic lesions begin to collapse from the early stage of the disease [28], our study for all lesions may represent similar characteristics.

We evaluated the relationship between the Total ExVol/Normal ExVol and the respiratory function, representing the ratio of how much of the whole lung had to be inflated to inflate the normal lung area. When the diseased area does not change in volume during respiration, the Total ExVol/Normal ExVol is almost 1, and in that case, the pathological significance of the diseased area is simply volume loss, such as atelectasis or partial resection of the lung. However, a higher value of this ratio may indicate a higher respiratory effort, as it implies a greater amount of diaphragmatic movement to inflate the normal lung area. Theoretically, two patients with identical inspiratory CT images could have different Total ExVol / Normal ExVol ratios if the expiratory CT images differed (Fig. 3B). These mechanical differences may be hidden in the background on routine inspiratory CT. As a result, the values were significantly higher in the DLCO%-predicted abnormal group than normal group (p = 0.0073). This fact that “DLCO%-predicted are lower in cases that require larger expansion of the total lung to inflate the normal lung” may play an important role in restrictive lung disease. Since it is speculated that the expansion of the hypofunctional area contributes less to gas exchange than normal, part of the diaphragmatic movement and inspiratory effort is spent on ineffective expansion. The diseased area may act as a negative buffer against respiration, not just a volume reduction.

Several limitations associated with the present study warrant mention. First, this was a retrospective study, and the number of cases was small. In most cases, expiratory CT is performed to evaluate airflow obstruction, and is not routinely performed to evaluate PF-ILD. In addition, patients undergoing expiratory CT are most likely to have other coexisting respiratory and cardiovascular abnormalities. To evaluate the characteristics of pure PF-ILD, these patients had to be excluded. It must be said that the statistical power is insufficient due to the small number of cases to try to evaluate its significance in relation to respiratory function.

In addition, all of the values measured in this study may be greatly influenced by the distribution of the lesions. To assess the impact of the distribution, it would be desirable to collect cases with various lesion volumes and types of distribution and check the effects on the measured values, but such a sufficient verification has not been conducted due to the small number of cases. Second, the pathologic diagnosis of PF-ILD pneumonia was not available, and the CT image patterns was mixed. Owing to the small number of cases, we were not able to evaluate each of these CT image patterns. Finally, the exclusion criteria allowed for cases of mild emphysema to be included. This was due to the difficulty in selecting only cases with no evidence of emphysema at all. Based on previous reports [21, 22, 29], it is possible that emphysema may have affected the FVC measurements in particular, and we may not have detected the exact relationship between the values we measured and FVC or FVC%-predicted.

In conclusion, although the diseased lung in PF-ILD is reduced the function and volume, the diseased lung retained expansion capacity comparable to the normal lung. This may be a characteristic of volume reduction in PF-ILD and may have a negative impact on respiratory function.
